# Specific expression of Kcna10, Pxn and Odf2 in the organ of Corti

**DOI:** 10.1016/j.gep.2012.03.001

**Published:** 2012-05

**Authors:** Francesca A. Carlisle, Karen P. Steel, Morag A. Lewis

**Affiliations:** Wellcome Trust Sanger Institute, Wellcome Trust Genome Campus, Hinxton, Cambridge CB10 1SA, UK

**Keywords:** Odf2, Cenexin, Pxn, Kcna10, Inner ear

## Abstract

The development of the organ of Corti and the highly specialized cells required for hearing involves a multitude of genes, many of which remain unknown. Here we describe the expression pattern of three genes not previously studied in the inner ear in mice at a range of ages both embryonic and early postnatal. Kcna10, a tetrameric Shaker-like potassium channel, is expressed strongly in the hair cells themselves. Odf2, as its centriolar isoform Cenexin, marks the dendrites extending to and contacting hair cells, and Pxn, a focal adhesion scaffold protein, is most strongly expressed in pillar cells during the ages studied. The roles of these genes are yet to be elucidated, but their specific expression patterns imply potential functional significance in the inner ear.

## Highlights

► We studied the expression of Pxn, Kcna10 and Odf2 in the developing mouse inner ear. ► We covered several ages between E14.5 and P5, and also looked at adults. ► Pxn is a focal adhesion protein expressed strongly in pillar cells. ► Kcna10 is a potassium channel expressed in hair cells. ► Odf2 (Cenexin) marks dendrites extending to and contacting hair cells.

## 1 Introduction

Hearing in mammals requires a finely tuned set of structures designed to detect sound waves and faithfully transmit information from those waves to the brain, where the information can be processed to perceive sound. Consequently, the inner ear contains a multitude of different cell types, particularly in the organ of Corti. Perhaps the best-known are the hair cells, responsible for converting sound waves into transmissible impulses. However, the supporting cells around them are also critical for hearing: they generate an environment in which the hair cells can function properly. All of these highly differentiated cells perform specialized functions, requiring the expression of unique combinations of genes. So far, at least 130 loci have been identified as affecting hearing in humans, including 50 known genes (Van Camp and Smith, Hereditary Hearing Loss Homepage, http://hereditaryhearingloss.org, reviewed in Dror and Avraham, 2009). However, many genes involved in hearing remain to be identified – as those remaining 80 loci without known genes indicate. It is possible that many of these ‘unknown’ genes have already been studied, but their expression in the ear or involvement in hearing has simply never been tested, and without prior evidence it is difficult to select one gene from many as a candidate for further study.

Presented here are three genes, *Pxn*, *Odf2* and *Kcna10*, which show specific expression in the mouse organ of Corti but which have not until now been identified as potentially important for hearing. *Odf2* and *Kcna10* were found to be expressed in the ear during a study of the effects of the microRNA miR96 on hearing in mice; *Kcna10* is downregulated in mice mutant for *Mirn96*, and *Odf2* is a potential target for the microRNA. Both showed striking expression patterns in the organ of Corti when tested by immunohistochemistry, but a thorough, individual examination of their temporal and spatial expression in the inner ear was not within the remit of that study (Lewis et al., 2009). *Pxn*, like *Kcna10*, was identified during an expression screen. These three genes were therefore chosen for a more in-depth examination of their expression in the inner ear.

The protein encoded by *Pxn* (*paxillin*) is a focal adhesion scaffold protein (Turner et al., 1990; reviewed in Deakin and Turner, 2008). It contains five leucine-aspartate (LD) motifs, four LIM domains, one SH3- and three SH2-binding domains, and a variety of phosphorylation sites (Brown et al., 1996; Salgia et al., 1995; Tong et al., 1997; Webb et al., 2005). Together these motifs and domains provide a number of sites on Pxn with which other proteins can interact. Integrin signalling seems indirectly to recruit Pxn to nascent focal adhesions through phosphorylation of Pxn’s third LIM domain (Brown et al., 1996; reviewed in Brown and Turner, 2004 and Parsons et al., 2010). At the focal adhesion, Pxn’s phosphorylation state and which proteins are bound to it at which sites play an important role in focal adhesion dynamics, and how the adhesion interacts with the cytoskeleton (Digman et al., 2008; reviewed in Deakin and Turner, 2008).

The *Pxn* gene is located on mouse chromosome 5, very close to the *Msi1* gene which is also expressed in supporting cells in the organ of Corti in adult mice (Murata et al., 2004). The human homologue, *PXN*, is located in the region of 12q24.23–12q24.31, which is within three deafness loci. The gene responsible for DFNA25 has been identified as *SLC17A8* (Ruel et al., 2008), and the gene responsible for DFNA64 is *DIABLO* (Cheng et al., 2011). The third locus, DFNA41, for which the causative gene has not been found, has been refined to 12q24.33, and is no longer near *PXN* (Yan et al., 2005).

*Odf2* (*outer dense fibre protein 2*) encodes two distinct proteins: the smaller, testis-specific structural protein Odf2, and the ubiquitous centriolar protein Cenexin (Brohmann et al., 1997; Hoyer-Fender et al., 2003; Huber and Hoyer-Fender, 2007; Huber et al., 2008; Nakagawa et al., 2001). Cenexin itself exists in a number of isoforms, although all contain the critical exon 3b, which Odf2 does not (Hoyer-Fender et al., 2003; Huber and Hoyer-Fender, 2007; Huber et al., 2008). In interphase Cenexin localizes to the appendages protruding from the mature centriole in the microtubule organizing centre, and indeed is required for appendage formation (Ishikawa et al., 2005; Lange and Gull, 1995; Nakagawa et al., 2001). Cenexin is also required to nucleate a primary cilium from the mature centriole, and once that occurs is found in the basal body and sometimes in the cilium itself (Ishikawa et al., 2005; Rosales et al., 2010; Schweizer and Hoyer-Fender, 2009). During cell division, Cenexin interacts with polo-like kinase 1 at the spindle poles to ensure proper chromosome segregation (Soung et al., 2006; Soung et al., 2009). Cenexin therefore is required for centriole function and chromosome segregation.

Mouse *Odf2* is on chromosome 2, and its human homologue *ODF2* is in 9q34.11. No deafness loci whose responsible genes are unknown cover this region (Van Camp and Smith, Hereditary Hearing Loss Homepage, http://hereditaryhearingloss.org, February 2012). The DFNB31 region is defined as 9q32-q34, but the gene involved has been identified as *WHRN* (Mburu et al., 2003).

The protein derived from *Kcna10* (*potassium voltage-gated channel subfamily A member 10*, also known as K_v_1.8) is a tetrameric Shaker-like potassium channel, with a voltage sensor region and a putative nucleotide binding region (Yao et al., 1995). Kcna10 is regulated in part by the soluble β subunit Kcna4b, which increases overall Kcna10 current and is currently thought to mediate upregulation of Kcna10 activity by cAMP and downregulation by cGMP (Lang et al., 2000; Tian et al., 2002). Expression of Kcna10 has been noted in the brain, aorta, kidney and heart; in the latter two it is believed to contribute to membrane potential stabilization (Lang et al., 2000; Tian et al., 2002; Yao et al., 1995, 1996, 2002).

In the mouse, *Kcna10* is on chromosome 3, and the human *KCNA10* gene is in 1p13.3, which is covered by DFNB82, for which the responsible gene is *GPSM2* (Walsh et al., 2010). No deafness loci which lack an associated gene cover this region (Van Camp and Smith, Hereditary Hearing Loss Homepage, http://hereditaryhearingloss.org, February 2012).

The present study describes the expression of these genes in the inner ear of wildtype mice at a range of ages around birth and at 9 weeks old. This analysis has revealed some striking expression patterns and provides new markers with which to follow innervation of the hair cells and markers for root cells, and suggests some possible roles for these molecules in auditory function.

## 2 Results

### 2.1 Pxn

**Fig. 1:**
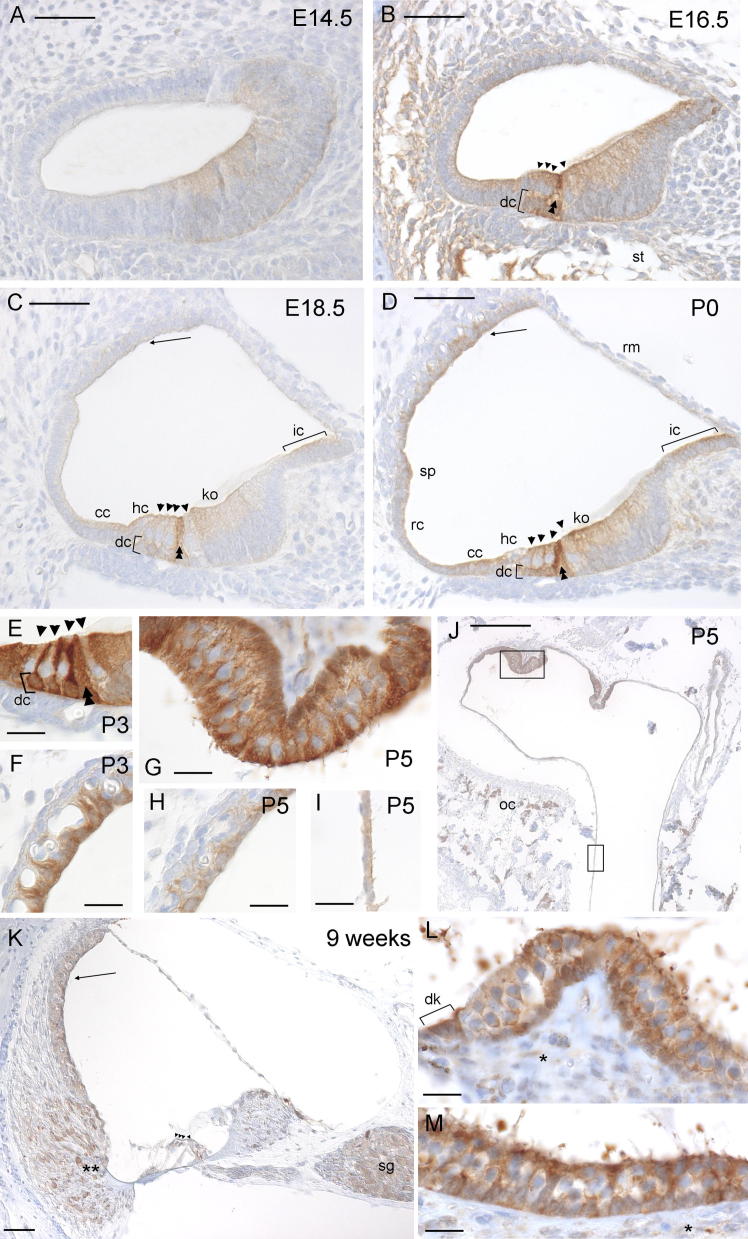
Immunohistochemistry for *Paxillin* expression in the mouse inner ear. Brown indicates positive staining. (A) Cochlear duct at E14.5, showing discrete staining in non-sensory patches. (B) Cochlear duct at E16.5, showing staining where the scala tympani is opening up and in supporting cells, especially the pillar and Deiters’ cells. (C) Cochlear duct at E18.5, showing staining in the pillar cells, interdental cells, Kölliker’s organ, cells of Claudius, Deiters’ cells, and faintly in the stria vascularis. (D) Cochlear duct at P0, showing staining in the pillar cells, Deiters’ cells, interdental cells, Kölliker’s organ, spiral prominence, Hensen cells, cells of Claudius, a single layer of the Reissner’s membrane, root cells and hair cells. (E) Detail of the hair cells at P3, showing heavy staining in the pillar cells, Deiters’ cells, Hensen cells and cells of Claudius. (F) Detail of the stria vascularis at P3, showing heavy staining in the marginal cells. (G) Detail of the crista at P5, showing heavy staining of supporting and hair cells. (H) Detail of the stria vascularis at P5, showing moderate staining in the marginal cells. (I) Detail of the vestibular duct lining at P5, showing moderate staining. (J) One crista and the common crus at P5, showing staining in supporting cells of the crista and cells lining the vestibular duct. Discrete cells in the otic capsule also show heavy staining. (K) Cochlear duct at 9 weeks old, showing staining in root cell processes, stria vascularis and spiral ganglion. (L) Crista at 9 weeks old showing staining in hair and supporting cells, and also the dark cells. There is faint expression in the neural dendrites. (M) Macula at 9 weeks old showing staining in hair and supporting cells, and faint expression in neural dendrites. For images A–D and K, bar indicates 50 μm. For images E–I, L and M, bar indicates 20 μm. For image J, bar indicates 200 μm. Abbreviations: dc, Deiters’ cells; ic, interdental cells; ko, Kölliker’s organ; sp, spiral prominence; hc, Hensen cells; cc, cells of Claudius; rc, root cells; rm, Reissner’s membrane; st, scala tympani; oc, otic capsule; sg, spiral ganglion; dk, dark cells. Stria vascularis is indicated by an arrow, hair cells by arrowheads, pillar cells by double arrowheads, neural dendrites by asterisks and root cell processes by double asterisks.

Pxn expression was cytoplasmic at all stages, and was never seen in the nuclei. Staining was visible in the organ of Corti from E14.5, in patches of tissue at the modiolar side of the cochlear duct (Fig. 1a). By E16.5, some expression of Pxn could be seen in most supporting tissues, particularly the Deiters’ cells and pillar cells, as well as below the basilar membrane where the scala tympani was opening up (Fig. 1b), but expression in the organ of Corti became much clearer at E18.5 when some expression also could be seen in all epithelial cells lining the cochlear duct (Fig. 1c). By far the strongest expression at E18.5 was, however, in the pillar cells (Fig. 1c). In all of these locations, a gradient of intensity was seen from the basal turn of the cochlea to the apex, with strongest expression at the base. This gradient remained in place through P0, although expression in all locations intensified (Fig. 1d). Expression was also noted in the inner layer of Reissner’s membrane, and to have intensified significantly in the marginal cells at P0. At P3, the general expression pattern was identical to that seen at P0, although expression in the pillar, Deiters’ and marginal cells had increased (Fig. 1e and f), and the root cell processes were showing moderate expression of Pxn (data not shown). However, at P5 expression levels in the marginal cells had decreased (Fig. 1h). All other expression levels and patterns remained the same as that seen at P3 (data not shown). In addition, at P3 and P5 discreet patches of Pxn expression could be seen in the otic capsule, perhaps marking developing osteocytes (Fig. 1j; Vatsa et al., 2008). In the vestibular system, Pxn expression was much simpler: heavy expression was seen in supporting cells, with expression also in the hair cells; expression levels generally increased from E16.5 through P5 (Fig. 1g and j). From E18.5 expression was also noted in cells lining the vestibular ducts, particularly the common crus (Fig. 1i and j). In adult mice, Pxn expression was strongest in the stria vascularis, root cell processes and the spiral ganglion. It was also present in the hair cells of the maculae and cristae, with marked expression in the dark cells adjacent to the crista (Fig. 1k–m).

### 2.2 Odf2

**Fig. 2:**
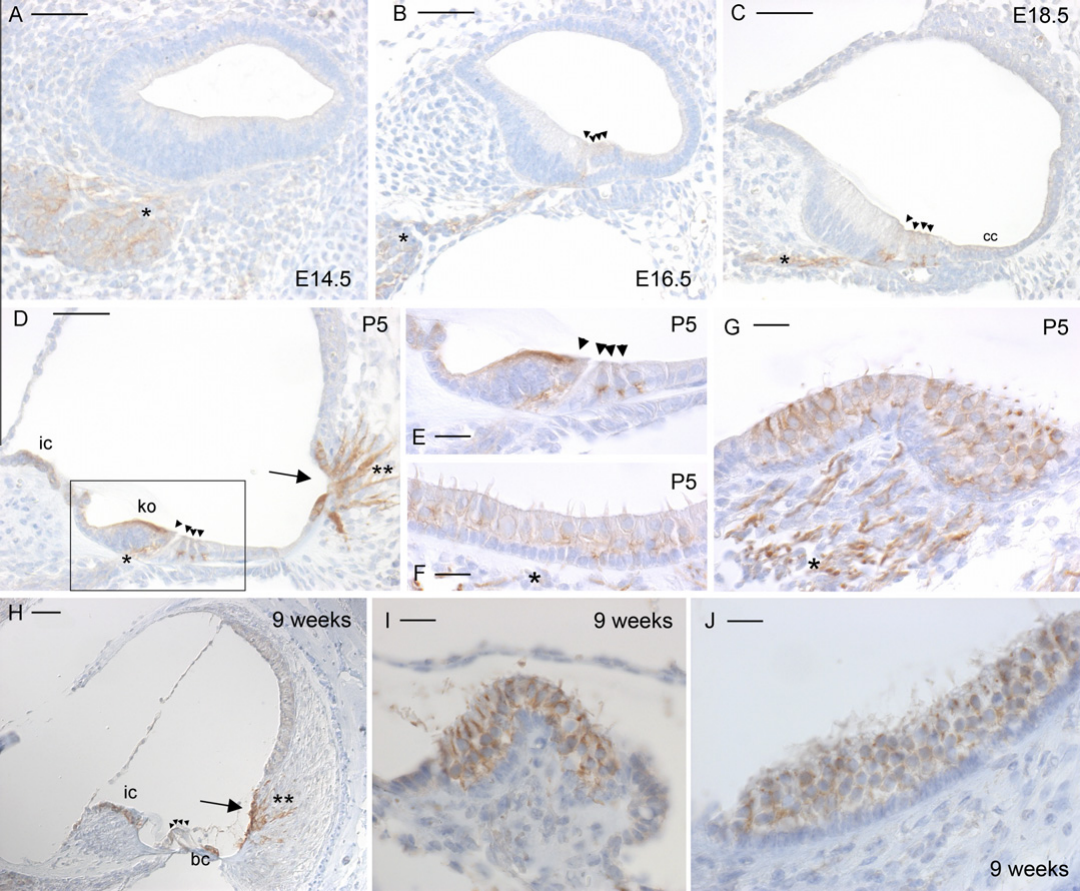
Immunohistochemistry for *Odf2* expression in the mouse inner ear. Brown indicates positive staining. (A) Cochlear duct at E14.5, showing clear staining of neurons. (B) Cochlear duct at E16.5, showing faint staining of the hair cells and stronger staining of neural dendrites. (C) Cochlear duct at E18.5, showing faint staining of the hair cells and cells of Claudius, plus marked staining of neural dendrites. (D) Cochlear duct at P5, showing strong staining of interdental cells, root cells and processes, the apical region of Kölliker’s organ, and neural dendrites. (E) Detail of hair cells at P5, showing strong staining of dendrites innervating the hair cells. (F) Macula at P5, showing faint staining of hair cells plus staining of dendrites. (G) Crista at P5, showing strong staining of hair cells and dendrites. (H) Cochlea at 9 weeks old, showing staining in interdental cells, Boettcher cells, root cells and root cell processes. (I) and (J) Crista (I) and macula (J) at 9 weeks old showing staining in vestibular hair cells. For images A–D and H, bar indicates 50 μm. For images E–G, I and J, bar indicates 20 μm. Abbreviations: cc, cells of Claudius; bc, Boettcher cells; ko, Kölliker’s organ; ic, interdental cells. Neurons are indicated by asterisks, hair cells by arrowheads, root cells by an arrow, and root cell processes by double asterisks.

Expression of Odf2 showed a marked difference between sensory regions in the organ of Corti and in the vestibular system. In the organ of Corti, neural tissue showed clear Odf2 expression right from E14.5 (Fig. 2a). From E14.5 to E18.5, Odf2 expression marked the neural dendrites as they extended to innervate first the inner and then the outer hair cells, as can be followed in Fig. 2a–c. At E18.5 and subsequent ages, the strongest Odf2 expression was in the portions of dendrites directly underneath hair cells, presumably at synapses. Hair cells were faintly stained from E16.5 (Fig. 2b), and the cells of Claudius from E18.5 (Fig. 2c). At P0, root cells showed faint expression as well (data not shown). The root cells and the root cell processes showed strong expression at P3 (data not shown) and P5 (Fig. 2d). At P5, strong expression could also be seen in the apical region of Kölliker’s organ, and moderate expression in the interdental cells (Fig. 2d). The strongest expression was still in the dendrites, though (Fig. 2e). However, at all ages hair cells in the organ of Corti showed only very weak expression of Odf2 (Fig. 2). In contrast, vestibular hair cells in the cristae showed stronger Odf2 expression. This began at E16.5, but was strongest at P5 (Fig. 2g). Hair cells in the maculae did not show anywhere near the same strength of expression, as Fig. 2f demonstrates. As in the cochlea, neurons in the vestibular system showed strong expression of Odf2 (Fig. 2f and g). Like Pxn, Odf2 expression was cytoplasmic. In adult mice, Odf2 expression was marked in the interdental cells, Boettcher cells, the root cells and root cell processes. Fainter expression was also visible in the hair cells of the maculae and cristae (Fig. 2h–j).

### 2.3 Kcna10

**Fig. 3:**
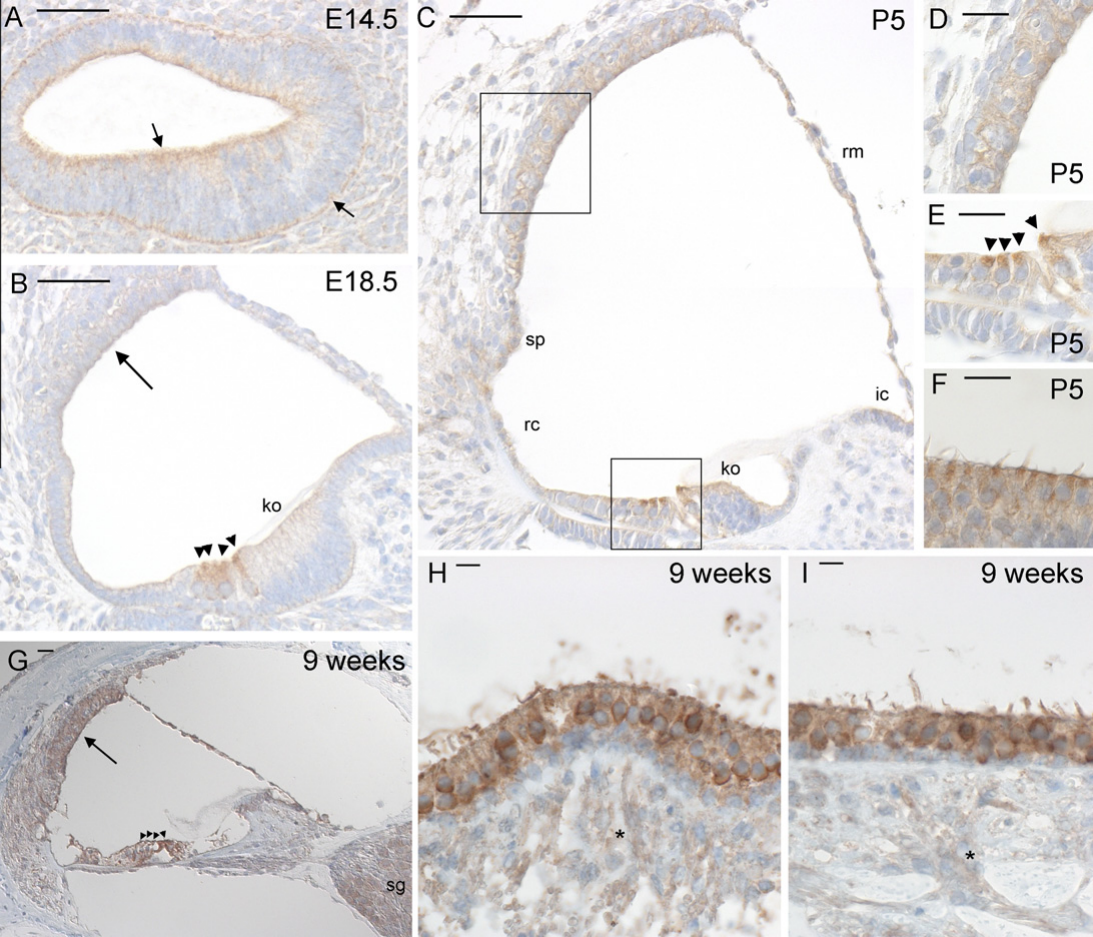
Immunohistochemistry for *Kcna10* expression in the mouse inner ear. Brown indicates positive staining. (A) Cochlear duct at E14.5, showing clear staining lining the interior and exterior of the cochlear duct. (B) Cochlear duct at E18.5, showing moderate staining of the hair cells and faint staining in Kölliker’s organ and stria vascularis. (C) Cochlear duct at P5, showing strong staining of the hair cells, moderate staining in the stria vascularis, spiral prominence and root cells and faint staining in Kölliker’s organ, interdental cells and Reissner’s membrane. (D) Detail of stria vascularis at P5, showing moderate staining. (E) Detail of hair cells at P5, showing strong staining in all hair cells. (F) Crista at P5, showing staining of the hair cells. (G) Organ of Corti at 9 weeks old, showing staining around the cochlear duct, most notably in stria vascularis and hair cells, and in the spiral ganglion. (H) and (I) Crista (H) and macula (I) at 9 weeks old showing strong staining in hair cells, supporting cells and fainter staining in neurons (asterisks). For images A–C, bar indicates 50 μm. For images D–G, bar indicates 20 μm. For images H and I, bar indicates 10 μm. Abbreviations: ko, Kölliker’s organ; sp, spiral prominence; rc, root cells; ic, interdental cells; rm, Reissner’s membrane; sg, spiral ganglion. Neurons are indicated by asterisks, hair cells by arrowheads. Arrows in panel A indicate staining, arrows in B and G indicate the stria vascularis.

At E14.5, moderate expression of Kcna10 lined the lumen of the cochlear duct and the basal lining of the duct, as seen in Fig. 3a. Some expression could also be seen in neurons (data not shown); all Kcna10 expression was cytoplasmic. Hair cells in the organ of Corti and the vestibular system began expressing *Kcna10* at E16.5 (data not shown). By E18.5, expression was largely limited to the hair cells, stria vascularis, and Kölliker’s organ, although some expression could be seen in other supporting cell types (Fig. 3b). Hair cells showed the highest expression levels. This pattern was continued through P3 (data not shown). At P5, hair cells still showed by far the strongest expression of Kcna10, particularly the apical domains of outer hair cells in the organ of Corti (Fig. 3e). The marginal and intermediate cells in the stria vascularis also showed moderate expression (Fig. 3d), as did the spiral prominence and root cells (Fig. 3c). Furthermore, very faint expression could sometimes be detected in the Reissner’s membrane, interdental cells, and Kölliker’s organ; at this stage all cells lining the cochlear duct appear to express Kcna10 at varying levels. In the vestibular system, hair cells seemed to show heavy expression of Kcna10 at P5, and supporting cells showed mild expression (Fig. 3f). This was consistent with expression in the vestibular system from E16.5 (data not shown). In the adult organ of Corti, Kcna10 is expressed in almost all cells lining the cochlear duct, and also in the spiral ganglion (Fig. 3g), while in the vestibular system hair and supporting cells express Kcna10 and expression is also seen in neurons (Fig. 3h and i).

## 3 Discussion

In this study, we present some unexpected and specific expression patterns for Pxn, Odf2 and Kcna10 in the mouse cochlea from E14.5 through P5 inclusive. These results are summarized in Table 1, and indicate that all three genes are expressed in both the organ of Corti and the vestibular system, albeit in different locations at different ages. Interestingly, all three genes show a much more stable expression pattern in the vestibular system. While expression in the cochlea underwent a variety of alterations between E14.5 and P5, expression in the vestibular system seemed to take shape around E16.5 and despite a general increase in expression the pattern did not alter much. This may be due to the earlier maturation of the vestibular system as a whole compared to the organ of Corti. It is worth noting that the limitations of immunohistochemistry mean this method can only provide a guide to locations of expression and some idea of relative expression levels. However, the current study does describe some specific and intriguing expression patterns for the genes studied.

The Pxn protein has been implicated in tight junction formation (Crawford et al., 2003) and this may explain the expression of Pxn in all cells lining the cochlear duct from E14.5 to P0, particularly at E18.5 (Slepecky, 1996).

Although Pxn expression is visible throughout the cochlear duct, at pre- and early post-natal stages it is always most marked in the pillar cells, and thus may serve as a pillar cell marker, similar to genes such as Prox1 and Cdkn1b, both of which are expressed in the developing organ of Corti before hair cell differentiation and are restricted to supporting cells, including pillar cells, by E16.5 (Bermingham-McDonogh et al., 2006; Chen and Segil, 1999).

Odf2 showed particularly high levels of expression in neurons and vestibular hair cells. Cenexin, the somatic *Odf2* product, is a centriolar protein required for centriole maturation, formation of the basal body and generation of the primary cilium, as well as for proper chromosome segregation during cell division (Ishikawa et al., 2005; Lange and Gull, 1995; Nakagawa et al., 2001; Schweizer and Hoyer-Fender, 2009; Soung et al., 2006). Therefore, all cells should express *Odf2* but typically at levels too low for detection by immunohistochemistry. Vestibular hair cells retain their kinocilium, which may explain the consistently high Odf2 expression seen in cristae hair cells.

More interesting, however, was the heavy expression of Odf2 in neural dendrites at pre- and early post-natal stages. Cenexin is known to associate with acetylated microtubules, which are found not only in centrioles but also forming a small number of defined, relatively stable paths in the cytoplasm along which organelles such as the mitochondria and endoplasmic reticulum can preferentially move (Donkor et al., 2004; Friedman et al., 2010; Huber et al., 2008). The high levels of Odf2 expression in neurons, root cells, root cell processes, and perhaps even the slightly elevated expression in hair cells might be due to higher levels of acetylated microtubules.

Known markers for organ of Corti dendrites and synapses include GluR2/3, Bassoon, Ctbp2 (Ribeye) and Neurofilament (Liberman et al., 2011; Kujawa and Liberman, 2009; Khimich et al., 2005; Hasko et al., 1990). Ctbp2 and Bassoon mark presynaptic ribbons of hair cells (tom Dieck et al., 2005; Khimich et al., 2005; Kujawa and Liberman, 2009) and GluR2/3 marks postsynaptic densities on the neurons (Liberman et al., 2011). Odf2 is most similar to Neurofilament, which is expressed in spiral ganglion neurons both pre- and post-natally, and is also transiently expressed in hair cells shortly after birth (Hasko et al., 1990).

The potassium channel encoded by *Kcna10* is voltage-gated and regulated at least in part by cyclic nucleotides (Lang et al., 2000; Tian et al., 2002; Yao et al., 1995, 2002). Kcna10 has already been proposed to play a role in stabilizing membrane potential in the proximal tubule of the nephron and in the heart (Lang et al., 2000; Tian et al., 2002; Yao et al., 2002). This immediately suggests it could be playing a similar role in the auditory and vestibular hair cells. Expression in the stria may support the stria’s role in potassium recycling, as potassium ions must be taken up from the perilymph by fibrocytes of the spiral ligament, and transferred through basal cells to intermediate cells, then via the intrastrial fluid to the marginal cells where it is finally released into the endolymph through the Kcnq1/Kcne1 transporters (reviewed in Hibino and Kurachi, 2006; Wangemann, 2006; Zdebik et al., 2009). Kcna10 expression was also evident in the root cells, which are also known to play a role in potassium recycling (Jagger et al., 2010); this would support such a model.

Kcna10 is most strikingly expressed in the hair cells, for which many markers are known, and its early expression is similar to that of both Atoh1 and Myo7a. Atoh1 is expressed first at E13, in the sensory epithelium from which hair cells will arise. By E17, its expression is restricted to the hair cells, and by P3, its expression is downregulated (Lanford et al., 2000). Myo7a has a very similar pattern of expression both temporally and spatially, but it is not downregulated at P3 (Sahly et al., 1997). At adult stages, however, Kcna10 expression is no longer specific to hair cells.

These hypotheses, drawn as they are from initial expression patterns, can however be no more than interesting starting points. Knockouts may well prove helpful in shedding light on the functions of these three genes in the ear. A knockout of *Odf2* has already been generated, but heterozygotes showed overall normal phenotypes and homozygous blastocysts failed even to implant in the uterine lining (Salmon et al., 2007). Hearing in the heterozygotes was not studied. A *Pxn* knockout has also been created, but again was homozygous lethal and the heterozygotes’ hearing was not evaluated (Hagel et al., 2002). A knockout for *Kcna10* has yet to be reported. It will be interesting to see, as work progresses on these genes, which ones have completely unique roles in audition and/or balance, and which prove redundant.

## 4 Experimental procedures

### 4.1 Sample collection and preparation

Wildtype mice from the C57BL/6J-Tyr^c-Brd^ strain were used for the expression study. Mated female mice were checked daily for the presence of a plug; the day of plug discovery was deemed embryonic day 0.5. Embryonic samples were collected at E14.5, E16.5, and E18.5 and postnatal pups were collected at P0, P3 and P5. The 9-week-old mice were from a mixed background of C57BL/6J-Tyr^c-Brd^ and C57BL/6N. The heads for all samples were then bisected, fixed for two days in 10% formalin at 4 °C, washed, dehydrated and cleared with xylene, then embedded in paraffin wax. The half heads from the 9-week-old mice were decalcified for two weeks in 10% EDTA after washing and before dehydration.

All experiments were carried out in full compliance with UK Home Office requirements.

### 4.2 Immunohistochemistry

Embedded samples were cut into 8 μm thick sections along the sagittal plane. Immunohistochemistry was then carried out on slides using the Ventana Discovery machines (Ventana Roche, Tucson, AZ, USA) with the manufacturer’s reagents (CC1 (Cat. No. 950-124), EZPrep (Cat. No. 950-100), LCS (Cat. No. 650-010), RiboWash (Cat. No. 760-105), Reaction Buffer (Cat. No. 95-300), and RiboCC (Cat. No. 760-107) and according to the manufacturer’s instructions. The DABMap™ Kit (Ventana; Cat. No. 760-124) with hematoxylin counterstain (Cat. No. 760-2021) and bluing reagent was used. For each gene, slides covering the entire inner ear for three different mouse samples at each age were stained, and the observed expression patterns were only considered reliable if present in all three samples. The primary antibodies used were as follows: Abcam (Cambridge, Cambs, UK) rabbit polyclonal to mouse Paxillin (ab2264), Abcam rabbit polyclonal to human Cenexin/ODF2 (ab43840; antibody raised against amino acids 750 to the end of the human protein, and therefore perhaps specific for Cenexin isoforms only), and LifeSpan BioSciences (Seattle, WA, USA) rabbit polyclonal antibody to human KCNA10 (LS-C31214). Secondary antibodies used were Jackson ImmunoResearch (West Grove, PA, USA) biotin-conjugated donkey anti-rabbit (711-065-152) and Jackson ImmunoResearch biotin-conjugated donkey anti-goat (705-065-147). All antibodies were diluted in ‘Antibody staining solution’: 10% foetal calf serum, 0.1% Triton, 2% BSA and 0.5% sodium azide in PBS.

Controls were run for secondary antibodies, wherein the above immunohistochemistry protocol was used on slides at each age but the primary antibody was omitted. For both secondaries, some staining was observed in certain patches of brain tissue, but none at all in the cochlea or vestibular system. Stained slides were examined and images obtained using an AxioCam HRc camera mounted on a Zeiss microscope. Images were then processed in Photoshop CS2.

## Figures and Tables

**Table 1:** Summary of expression patterns seen for Pxn, Odf2 and Kcna10 in the cochlea at E14.5, E16.5, E18.5, P0, P3, P5 and 9 weeks.

	Age	Cochlear duct	Otic capsule	Dendrites	Neurons	Hair cells	Pillar cells	Deiters’ cells	Hensen cells	Cells of claudius	Boettcher cells	Interdental cells	Kölliker’s organ	Root cells	Root cell processes	Scala tympani	Spiral prominence	Marginal cells	Stria vascularis	Reissner’s membrane	Spiral limbus
Pxn	E14.5	+																			
E16.5						+	+	+	+		+	+	+		+			+		
E18.5					+	+	+	+	+		+	+	+					+		
P0					+	+	+	+	+		+	+	+			+	+	+	Inner layer	+
P3		+			+	+	+	+	+		+	+	+	+		+	+	+	Inner layer	+
P5		+			+	+	+	+	+		+	+	+	+		+	+	+	Inner layer	
9 weeks				+										+			+	+		

Odf2	E14.5			+	+																
E16.5			+	+	+															
E18.5			+	+	+				+											
P0			+	+	+				+				+							
P3			+	+	+				+				+	+						
P5			+	+	+				+		+	Apex	+	+						
9 weeks										+	+		+	+						

Kcna10	E14.5	+			+																
E16.5	+			+	+															
E18.5				+	+				+		+	+	+			+		+	+	
P0				+	+		+	+	Apex		+	+	+			+		+	+	
P3				+	+				Apex		+	+	+			+		+	+	
P5				+	+						+	+	+			+	+	+	+	
9 weeks	+			+	+	+	+	+	+	+	+	+	+	+		+	+	+	+	
